# Word add-in for ontology recognition: semantic enrichment of scientific literature

**DOI:** 10.1186/1471-2105-11-103

**Published:** 2010-02-24

**Authors:** J Lynn Fink, Pablo Fernicola, Rahul Chandran, Savas Parastatidis, Alex Wade, Oscar Naim, Gregory B Quinn, Philip E Bourne

**Affiliations:** 1 Skaggs School of Pharmacy and Pharmaceutical Sciences, University of California, San Diego, CA, 92093-0444 USA; 2 External Research, MS 99/4618, Microsoft Corporation, 1 Microsoft Way, Redmond, WA, 98052 USA; 3 San Diego Supercomputer Center, 10100 Hopkins Dr., San Diego, CA, 92093-0743 USA

## Abstract

**Background:**

In the current era of scientific research, efficient communication of information is paramount. As such, the nature of scholarly and scientific communication is changing; cyberinfrastructure is now absolutely necessary and new media are allowing information and knowledge to be more interactive and immediate. One approach to making knowledge more accessible is the addition of machine-readable semantic data to scholarly articles.

**Results:**

The Word add-in presented here will assist authors in this effort by automatically recognizing and highlighting words or phrases that are likely information-rich, allowing authors to associate semantic data with those words or phrases, and to embed that data in the document as XML. The add-in and source code are publicly available at http://www.codeplex.com/UCSDBioLit.

**Conclusions:**

The Word add-in for ontology term recognition makes it possible for an author to add semantic data to a document as it is being written and it encodes these data using XML tags that are effectively a standard in life sciences literature. Allowing authors to mark-up their own work will help increase the amount and quality of machine-readable literature metadata.

## Background

In the current era of scientific research, efficient communication of information is paramount. Scientists are uncomfortably aware of the exponential growth of digital literature archives and the disproportionate growth of effective data-mining tools. It is currently a major effort in the bioinformatics community to automate the extraction of knowledge from literature [1,2]. Automated knowledge extraction is crucial for 21^st ^century research, especially as research is becoming increasingly more interdisciplinary, needs to be easier to navigate, needs to support the translation of natural language to information quanta, and needs to support data integration efforts [3-5]. In response, the nature of scholarly and scientific communication is changing; cyberinfrastructure is now absolutely necessary and new media are allowing information and knowledge to be more interactive and immediate [6,7].

While this revolution in scholarly communication has been imminent, the approach to dealing with it has not evolved at the same pace. Many basic tools to assist in knowledge extraction from literature already exist (such as cyberinfrastructure, electronic databases, ontologies, and machine-readable document standards), but the scientific community has yet to use them effectively on a large scale. The Semantic Web - an extension of the World Wide Web that enables more meaningful use of electronic resources via automated processes - is an ideal platform for these efforts [8-10], but there is a significant gap to be bridged between the providers and users of the information and the structure of the information. In a recent review Krallinger, Valencia, and Hirschmann nicely summarize the current challenges and resultant applications in the biological sciences which attempt to bridge this divide [11]. Ruttenberg et al. discuss the activities of the Semantic Web Health Care and Life Sciences Interest Group (HCLSIG) which aims to explore and enable the Semantic Web in biomedical domains [5].

One notable innovation is the creation and application of ontologies - specifications of entities, their attributes, and relationships to other entities in a defined domain. Ontologies underpin our efforts to translate natural language into quantized, standardized information. In the biological sciences, ontologies have attained so much popularity that it has been suggested that their proliferation is increasing in tandem with biological data [12,13]. Considering that the creation of an ontology can require years of work by a large team of experts, this popularity underscores the perceived importance of these efforts. The Gene Ontology in particular is currently widely used in the annotation of many of biological databases [14]. However, the reliable assignation of an ontology term to an entity in one of these databases necessitates a manual review by expert biocurators - a slow process and one that does not scale to the current level of research output [15].

A particularly advantageous use of ontologies is applying them to scientific literature in order to automatically identify, or infer, terms from one or more ontologies in the text of a document. Several groups have made significant contributions here, although every method has limited accuracy (see [1,2,15-21] for a few examples). Another challenge that is equally daunting is making these data available in the most useful and easily accessible way possible. Currently, the results from automated literature annotation projects are distributed over a number of databases and websites and there is no unified method of either storing or distributing these data. Two excellent approaches to resolve these issues, at least in part, have been undertaken by both authors and publishers. The Royal Society of Chemistry Publishing Group's Project Prospect^1 ^has semantically enriched all articles published in their journals in a machine-readable way. The project won the 2007 ALPSP/Charlesworth Award for Publishing Innovation, a strong indicator of community approval and interest because the judging panel represented not only publishers, but also scientists and librarians. A similar approach for a single article was undertaken by bioinformaticians in collaboration with the original article authors, and serves as an elegant example of how much can be gained by both semantic enrichment and author-assisted curation [22,23]. Both initiatives use their own mark-up syntax.

These projects illustrate the need for, and promise of, semantic enrichment, but there is a noticeable dearth of tools that assist authors in these efforts. Several exist, but have been developed for specific groups of users or very specific applications and are generally not publicly available for use or modification. A few others are available, such as the domain-agnostic Semantic MediaWiki extension^2 ^and WYSIWYM [24,25], and the biomedical-specific OnTheFly [26], but these lack ease of use, flexibility, extensibility, or do not allow for author-mediated curation.

As a community, we are certainly making progress in automated approaches for inferring and assigning semantic data in literature. However, this process will likely never be perfectly accurate or complete. There are three points that virtually all researchers interested in these efforts will agree upon: 1) adding semantic data to scientific articles is highly beneficial (indeed necessary for the Semantic Web path); 2) accurate and complete inference of these data without at least *some *human expert curation is not currently possible; and 3) accurate and complete inference of these data *after *the document has been made widely available is an intractable problem. To overcome these challenges, we must prevail upon the authors to assign semantic data to their articles *prior *to publication or distribution. The Word add-in presented here will assist authors in this effort using *community standards *and by making it possible for the author of the document, the absolute expert on the content, to do so *during the authoring process *and to *provide this information in the original source document*.

## Implementation

### Architecture

This software functions as an add-in for Microsoft Word 2007. It was developed using the .NET platform and can be installed via a Windows installer. The add-in relies on two key features of Word 2007, its default use of a XML based file format (Office Open XML, specified by ISO/IEC, IS 29500, and Ecma International, Ecma 376, international standards) and Word's extensibility, both at the user interface and file format levels. At runtime, the add-in generates and stores a configuration file on the end-user's system.

The add-in presents a custom ribbon, a new user interface element introduced in Word 2007, a side panel, and custom dialogs to interact with the end-user. It was a design goal to shield the author from having to be aware of the underlying file format or XML tags. Instead, the goal was to present a user experience that was as intuitive as possible, and that assisted the end-user in their task in a largely automated fashion. The add-in also relies on the Smart Tag architecture in Word, which enables actions to be presented to end-users based on text in the document being recognized through regular expressions or text matching.

The add-in contains knowledge of the National Center for Biomedical Ontology (NCBO) BioPortal [12,27] and three major biological databases: the Protein Data Bank (PDB), the UniProt Knowledgebase (UniProtKB) [28], and the NCBI databases GenBank and RefSeq [29,30]. When the end-user selects an ontology, the add-in downloads the ontology file via NCBO web services. The biological database identifiers are recognized via pattern matching.

### User Interface

#### Inline Recognition, Highlighting, and Mark-up of Informative Terms

**Figure 1:**
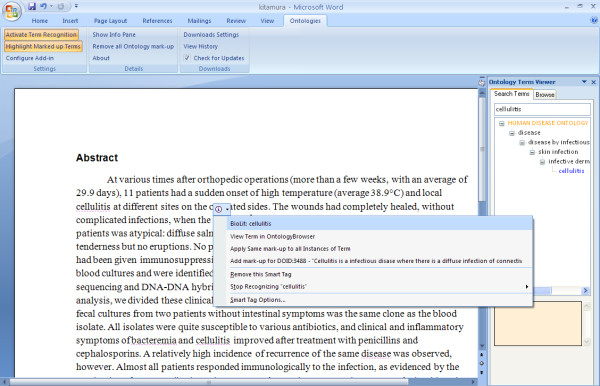
**Adding mark-up to a recognized term**. A SmartTag appears when a term is recognized. Clicking on the SmartTag brings up a menu of options for action including adding mark-up for the recognized term and all other instances of the termignoring all instances of this termignoring this particular instance of the termand further exploring this term within the context of the ontology it belongs toas seen in the InfoPane to the right of the document. The document shown hereand in Figure 2is excerpted from *J Clin Microbiol. 2007 Jan;45(1):31-8 *[54].

The add-in recognizes terms in the manuscript that belong to ontologies or databases selected by the user in the configuration panel (Figure 1). The recognition takes place automatically in the background, and recognized terms are marked with a dotted, purple underline. Hovering with the mouse over recognized terms presents a Smart Tag icon above the term. A user can then click on that Smart Tag to obtain a list of options for further action which include: add mark-up for this term, ignore this term, or view the term in the ontology browser (when applicable). The marked-up terms are made visible by choosing to highlight the terms; they will appear with a yellow background. Term recognition and highlighting can be toggled by clicking the "Activate Term Recognition" and "Highlight Marked-up Terms" buttons on the ribbon. Added mark-up will not be visible to the user, but will be saved in XML tags in the .docx file (see Table 1 for descriptions of the tags selected from the National Library of Medicine DTD). If a recognized term appears in more than one ontology, all instances of that term will be listed in the Smart Tag menu. The user can then decide which usage is most appropriate, if any, and select that instance. The add-in relies on the author's discretion to choose the single best term. Currently, overlapping tags are not allowed although the mark-up can be adapted for this particular use. More details describing the term recognition are available in the User's Guide at the CodePlex website.

**Table 1:** XML Tags

**Ontology Term Mark-up**

*General Format*

<named-content content-type="biolit" id="ncbo_id=X;term_id=Y:Z;term=Q;url=http://H"> Q </named-content>

*Example with data*

<named-content content-type="biolit" id="ncbo_id=38436;term_id=CL:0000031;term=neuroblast;url=http://bioportal.bioontology.org/ visualize/39004"> neuroblast </named-content>

*Note: *url=http://HHH*is optional*

**Database Identifier Mark-up**

*General Format*

<ext-link xlink:href="http://linktodatabase" ext-link-type="databaselabel"> DATABASEID </ext-link>

*Example with data*

<ext-link xlink:href=http://www.rcsb.org/pdb/explore/explore.do?structureId=1MU2 ext-link-type="pdb"> 1MU2 </ext-link>

Semantic data are marked up using tags from the NLM DTD http://dtd.nlm.nih.gov/. The <named-content/> and <ext-link/> tags are used for ontology terms and database identifiers, respectively.

Hovering over a marked-up term will produce a new Smart Tag menu option to mark-up all recognized instances of the same term with the same mark-up. The user can also choose to stop recognizing a term.

#### Built-in Knowledge of Ontologies and Databases

**Figure 2:**
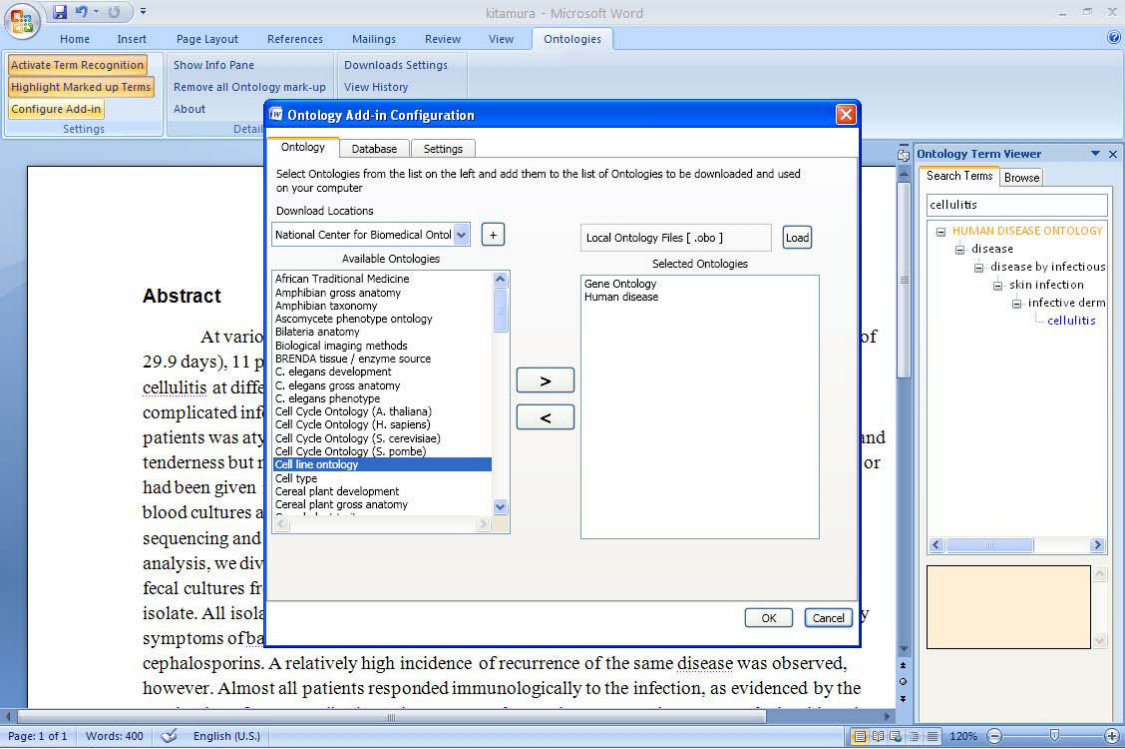
**Add-in configuration panel**. The configuration panel allows a user to download ontologies of interest from NCBO or provide custom ontologies from the local machine or a remote source. Database ID recognition can also be activated or deactivated via this panel.

The add-in provides a list of biomedical ontologies to download from NCBO and a list of databases for which ID recognition is possible (Figure 2). A user may also supply alternate OBO-formatted ontology files if an ontology is not already listed in the configuration panel. A user should select and download the ontologies that are most appropriate to the topic of the manuscript or document.

The add-in uses pattern matching to recognize database identifiers. When an identifier corresponding to one of the supported databases is typed, the add-in will identify the database(s) to which this identifier belongs and generate a Smart Tag with the options to mark-up or ignore the ID. The add-in uses the following regular expressions to match PDB, UniProtKB, and NCBI identifiers:

Protein Data Bank

/([1-9]][A-Z]{1}[A-Z\d]{2})/

UniProtKB

/[A-Z]{1}[0-9][A-Z][0-9A-Z]{2}[0-9]/

/[A|O|P|Q]{1}[0-9][0-9A-Z]{3}[0-9])/

NCBI (GenBank, RefSeq)

/[A|B|C|D|E|F|G|H|I|J|K|L|M|N|S|T|U|V|W|X|Y|Z]\d{5}/

/[A|B|C|D|E|F|G|H|I|J|K|L|M|N|S|T|U|V|W|X|Y|Z][A-Z]\d{6}/

/[A|B|C|D|E|F|G]A[A|E]\d{5}/

/[A|N|X|Y|Z][C|P|M|R|W|Z|S]\_\d{6,}/

Database identifiers are expected to be preceded by a space.

Ontologies may be added, updated, or removed via the configuration panel. Similarly, recognition of each database may be selected and unselected.

#### Custom Markup and the Ontology Browser

Ontology browse and search capabilities are available in the Info Pane. The browser allows a user to select an ontology and then navigate through it to view terms and their relationships. Alternatively, a user may search for the existence of a term. The Info Pane may be useful for finding terms that are not recognized, but probably should be, or for finding the context and definition of a term.

In the instances of terms that are semantically important, but are not recognized by an available ontology, an existing ontology term can be applied by right-clicking on the term of interest in the manuscript, navigating to the desired ontology term in the browser, and clicking "Mark-up Selection". For example, a user may use the term "hairy T cell leukemia", which will not currently be recognized; the equivalent ontology term "Leukemia, T-Cell, HTLV-II-Associated" can then be selected and applied while maintaining the original term in the manuscript text.

#### Interoperability

There are often situations in which a manuscript is shared among several authors. In the event that one author is not using the add-in, any XML added via the add-in by a different author is maintained although not displayed. It can be displayed if that author chooses to use the add-in later.

The expressivity of the NLM XML tags we have chosen to use is admittedly somewhat limiting and awkward for ontology terms, but we place a higher priority on conforming to existing standards with the expectation that the add-in will evolve with the community. For example, the representational model RDF is gaining in acceptance in the biomedical domain [5,31,32] and the Semantic Web Health Care and Life Sciences Interest Group (HCLSIG) is working with NLM to translate their resources to RDF [5].

#### Community Usage

The CodePlex site, which hosts the source code and compiled add-in, supports active discussions between users and developers and allows users to report bugs and request features. So far, the add-in has received interest from a wide range of bodies including repositories, academic groups, pharmaceutical companies, and electronic biological resources. In many of these cases, users have a custom ontology that they wish to use for a very specific application. There is at least one implementation of a parser for the marked-up .docx files. Some users have mentioned that they would prefer a simpler mark-up scheme. The add-in was initially developed for use with life sciences literature, hence the incorporation of the NLM XML tags. However, the open source code could easily be changed to use a different schema and generate an alternate add-in that may well be of broader use to the community and succeed the existing add-in in popularity.

We have not been apprised by users of their particular use cases, but we suggest some possibilities here. Publishers that are interested in strengthening electronic versions of their paper may request that authors use the add-in to mark-up database identifiers. Many life sciences publishers already require authors to use GenBank or PDB IDs, when applicable, in their manuscripts; the add-in would be the next step in automatically linking the IDs in the electronic paper to those databases. Some institutions may request that all internal documents conform to a controlled vocabulary, thus ensuring consistency and improved searching of archived documents.

## Results

The Word add-in for ontology term recognition makes it possible for an author to add semantic data to a document as it is being written and it encodes these data using XML tags that are effectively a standard in life sciences literature. Allowing authors to mark-up their own work will help increase the amount and quality of machine-readable literature metadata. The add-in recognizes terms simultaneously from several ontologies so that authors can select as many as they need in order to have the best coverage of their topic. When terms exist in these ontologies, but are not recognized by the add-in because they do not match the author's usage, the author can select the word or phrase of choice and explicitly apply the appropriate ontology term to that text. This enables semantic mark-up while maintaining the flexibility and nuance inherent in written language.

The add-in facilitates the identification of appropriate terms via the ontology browser. The browser allows an author to search an ontology with a word or phrase and, if a result is found, it displays the matches within the hierarchy of the ontology. This provides context for the terms within the ontology so the author can make a more informed evaluation of that term. The author can also navigate through the hierarchy to explore adjacent terms for more general or more specific concepts. It should be noted that this display is not entirely complete for terms with multiple parentage; only one parent is used in the hierarchy. NCBO has a more sophisticated search and visualization capability that an author can use if a concept is particularly complex. The author can visit the NCBO BioPortal website and search all ontologies with keywords. The results will show any matching terms and the ontologies they are members of; each term can then be explored within the context of its ontology. If a term belongs to an ontology that has not already been selected in the add-in, this ontology can then be added via the configuration panel. The term of interest can then be navigated to in the InfoPane and applied to the desired word or phrase in the document.

## Conclusions

The difficulties involved in any effort to add semantic mark-up are myriad and this add-in does not resolve all of them. While we think it is a significant step forward, it also highlights some of the more difficult challenges (see [33] for an illuminating discussion of these).

The use of ontologies is a solid initial step in defining what is effectively a controlled vocabulary for term recognition in natural language. These ontologies represent a vast amount of expertise and careful consideration across a wide range of domains. However, they were not created for automated term recognition so it is unsurprising that they are not a perfect fit for this application.

A desirable goal in the creation of an ontology is the inclusion of univocal terms - terms which are unambiguous and precise. For example, the Human Disease Ontology^3 ^contains the term "Leukemia, T-Cell, HTLV-II-Associated," which is very precise and descriptive, but is not likely to appear verbatim in a manuscript and, thus, is not likely to be recognized in a string or pattern matching approach. The ontology creators recognized that terms may have different usages, so most ontologies assign synonyms to the preferred usage of a term. These synonyms can be used in addition to the preferred term to increase the chance of successfully inferring a semantically important word. For example, the synonym for the aforementioned term, "Atypical hairy cell leukemia (disorder)," is a bit more natural and easier to automatically recognize, but actual papers that discuss this disease use "hairy cell leukemia", "hairy-cell leukemia", "hairy T cell leukemia", and "T cell hairy leukemia," terms that are not included in the ontology synonym list [34-38]. "Hairy cell leukemia" is a separate (less specific) term in this ontology, parent to "Leukemia, T-Cell, HTLV-II-Associated" but also to 12 other distinct leukemias.

There are occasions when it is not always desirable to use such precise terms when writing a manuscript. General concepts are often necessary, for example, the Human Disease Ontology term "leukemia." However, when a term is less precise it may have different conceptual meanings. The Human Disease Ontology and Family Health History Ontology [39] both contain the term "leukemia," but define the term alternately as a disease and a medical diagnosis - subtle, but potentially significant, distinctions. Although the add-in allows an author to associate any word or phrase with a specific ontology term, this requires an extra step by the author (at least once per document).

Rather than invent an ontology alternative to address these problems, a possible adaptation to existing ontologies might be the inclusion of an additional set of synonyms for a term that reflect its use *in natural language*. Automated finding of these types of synonyms in extant literature is feasible (if not entirely accurate) using heuristic approaches [40]. Synonyms found in this manner, or gathered from term-mapping databases [41-44], could be used as a supplement to the ontologies. Incorporating a more sophisticated term recognition approach such as term normalization or other heuristic rules (for example [45-49]), into the add-in would also likely be a significant improvement.

Regardless of the automated recognition approach, human disambiguation of terms and synonyms would still require some consideration by the author to ensure that the intended meaning is accurately conveyed. Even professional biocurators do not always agree on the most appropriate terms to assign to concepts in an article [50]. For an author who lacks familiarity with ontologies or literature curation, the process of trying to first identify the semantically important words and phrases in their manuscript and then the most appropriate term to use to describe them could prove to be too challenging, at least without clear guidelines from the intended manuscript recipient [51-53]. These difficulties may be magnified if co-authors of the manuscript disagree on term usage. Initiatives such as ODIE^4 ^show that establishing a feedback loop between ontology developers and ontology users frequently results in the discovery of new, relevant terms to add to existing ontologies. Ontology developers from the Gene Ontology, for example, have expressed keen interest in creating such a system within this add-in and we intend to explore this in a future version. Ideally, we would also like to be able to enable recognition and mark-up of relations between terms, but this is a significant challenge in its own right and is beyond the scope of the current project.

Although these challenges in the semantic enrichment of literature have not yet been resolved, we believe that the add-in is a significant advance and that it may provide the necessary stimulus to engage researchers beyond the bioinformatics community. Importantly, this add-in can work in concert with the Article Authoring add-in^5 ^which converts a .docx manuscript into the National Library of Medicine's XML format^6 ^- required for deposition of articles in PubMed Central and used by many life sciences publishers. The combined use of these add-ins would generate a document that maintains author-added semantic metadata and can be incorporated directly into these workflows without any further effort on the part of publishers or archives. Feedback during practical use from a broad and large user-base will help define any barriers to common use and will guide the design of an interface that can lower those barriers. Few of us want to spend yet more time and effort writing or typesetting papers, but if this effort culminated in a reference to the paper from a database or other resource, authors would likely be rewarded with an increased citation rate and wider readership, in addition to an overall improvement in the accessibility of knowledge.

## Availability and requirements

Download: The add-in and source code are publicly available at http://www.codeplex.com/UCSDBioLit. No registration, login, or material transfer agreement is required.

Requirements: Windows XP or Vista; Word 2007

License: Microsoft Public License (Ms-PL), an Open Source Initiative (OSI)-approved license^7^

## Competing interests

The authors declare that they have no competing interests.

## Authors' contributions

JLF coordinated the development work by UCSD and contributed to the design. RC was responsible for the majority of add-in development. GBQ prototyped the first version of the add-in. PEB conceived the idea of the add-in and provided direction on its development. PF, ON, AW contributed to the architecture and development. SP contributed to the initial design and concept. All authors read and approved the final manuscript.

## Appendix

^1 ^http://www.rsc.org/Publishing/Journals/ProjectProspect/

^2 ^http://semantic-mediawiki.org/wiki/Semantic_MediaWiki

^3 ^http://diseaseontology.sourceforge.net/

^4 ^http://www.bioontology.org/ODIE

^5 ^http://www.microsoft.com/downloads/details.aspx?FamilyID=09C55527-0759-4D6D-AE02-51E90131997E&displaylang=en

^6 ^http://dtd.nlm.nih.gov/

^7 ^http://www.opensource.org/licenses/ms-pl.html
